# Malignant hyperthermia testing in probands with NO adverse anesthetic reaction

**DOI:** 10.1186/1471-2253-14-S1-A2

**Published:** 2014-08-18

**Authors:** Carly Sterling, Henry Rosenberg, Natalia Kraeva, Sheila Riazi

**Affiliations:** 1 Malignant Hyperthermia Investigation Unit, Department of Anesthesia, Toronto General Hospital, M5G 2C4 Toronto, Canada; 2 Department of Anesthesia, Saint Barnabas Medical Center, Livingstone, 07039 New Jersey, USA

## Background

Malignant hyperthermia (MH) is characterized by an adverse reaction to volatile anesthetic, and /or succinylcholine. Typically, following an adverse anesthetic reaction or positive family history, patients will undergo caffeine-halothane contracture (CHCT) and/ or genetic testing. However, sometimes patients with no individual or family history of anesthetic reaction are referred for MH testing due to a variety of reasons. The objective of our study was to investigate reasons for referrals in non-anesthetic cases, and assess their phenotype.

## Materials and methods

Following institutional research ethics board approval, all the CHCT-tested probands at our MH center were identified. Patients with anesthetic reactions were excluded. Reasons for referrals, baseline CK, genetics results, histopathology were analyzed and compared between patients with positive and negative CHCT results. Response to dantrolene among patients with positive CHCT was also assessed. Wilcoxon rank sums test, and fisher’s exact test were used for numerical, and categorical parameters, respectively.

## Results

Between 1992-2012, 152 probands with no anesthetic reaction were identified. Of these, 104 (68.4%) had positive CHCT. Reasons for referrals included unexplained high creatine kinase-CK (50.6%), post-viral chronic fatigue (41.4%), post-exercise rhabdomyolysis (7.9%), and heat stroke (0.6%).

Fifty-nine patients with high CK (76.6%), and 36 patients with post-viral chronic fatigue (57.1%) had positive CHCT based on the standardized North American CHCT test protocol. The viral illness included influenza, Epstein-Barr, and cytomegalovirus. The fatigue was defined as muscle pain, weakness, and cramps, interfering with functional ability, lasted more than three months after the onset of viral illness.

Thirty-eight (36.5%) patients with positive CHCT had abnormal histomorphology, which included central cores, and multi-minicores. Three patients carried causative mutations in Ryanodine receptor-I (RYR-I); of these, 2 were referred for unexplained high CK, and 1 was referred for exercise-induced rhabdomyolysis. Forty patients with positive CHCT (38.4%) were given oral dantrolene, in which 30 (75.0%) responded with improvement of musculoskeletal symptoms, and reduction in CK.

## Conclusions

MH susceptibility as confirmed by the caffeine halothane contracture test may predispose patients to a variety of non anesthetic induced muscle abnormalities. The results support the contention that MH maybe more than an anesthetic disorder but should be interpreted in the context of the limitations of the sensitivity of the CHCT.

